# Tumor-Infiltrating Macrophages in Post-Transplant, Relapsed Classical Hodgkin Lymphoma Are Donor-Derived

**DOI:** 10.1371/journal.pone.0163559

**Published:** 2016-09-29

**Authors:** Genevieve M. Crane, Mark A. Samols, Laura A. Morsberger, Raluca Yonescu, Michele L. Thiess, Denise A. S. Batista, Yi Ning, Kathleen H. Burns, Milena Vuica-Ross, Michael J. Borowitz, Christopher D. Gocke, Richard F. Ambinder, Amy S. Duffield

**Affiliations:** 1 Department of Pathology, The Johns Hopkins Hospital, Baltimore, Maryland, United States of America; 2 Sidney Kimmel Comprehensive Cancer Center, The Johns Hopkins Hospital, Baltimore, Maryland, United States of America; United Arab Emirates University, UNITED ARAB EMIRATES

## Abstract

Tumor-associated inflammatory cells in classical Hodgkin lymphoma (CHL) typically outnumber the neoplastic Hodgkin/Reed-Sternberg (H/RS) cells. The composition of the inflammatory infiltrate, particularly the fraction of macrophages, has been associated with clinical behavior. Emerging work from animal models demonstrates that most tissue macrophages are maintained by a process of self-renewal under physiologic circumstances and certain inflammatory states, but the contribution from circulating monocytes may be increased in some disease states. This raises the question of the source of macrophages involved in human disease, particularly that of CHL. Patients with relapsed CHL following allogeneic bone marrow transplant (BMT) provide a unique opportunity to begin to address this issue. We identified 4 such patients in our archives. Through molecular chimerism and/or XY FISH studies, we demonstrated the DNA content in the post-BMT recurrent CHL was predominantly donor-derived, while the H/RS cells were derived from the patient. Where possible to evaluate, the cellular composition of the inflammatory infiltrate, including the percentage of macrophages, was similar to that of the original tumor. Our findings suggest that the H/RS cells themselves define the inflammatory environment. In addition, our results demonstrate that tumor-associated macrophages in CHL are predominantly derived from circulating monocytes rather than resident tissue macrophages. Given the association between tumor microenvironment and disease progression, a better understanding of macrophage recruitment to CHL may open new strategies for therapeutic intervention.

## Introduction

Lymph nodes involved by classical Hodgkin lymphoma (CHL) typically contain relatively few neoplastic Hodgkin/Reed-Sternberg (H/RS) cells, which are scattered throughout a mixed inflammatory background composed of lymphocytes, macrophages, neutrophils, eosinophils and plasma cells. The H/RS cells can comprise as little as 1% of the cellularity [1]. While only the H/RS cells are neoplastic, the composition of the inflammatory background correlates with prognosis and response to treatment [2–10]. It is unclear to what extent the H/RS cells themselves dictate the inflammatory environment.

Of particular interest are the tumor-associated macrophages and their source as increased numbers of tumor-associated macrophages correlate with a poor prognosis in patients with CHL [11–16]. While tissue macrophages had long been presumed to be derived from circulating monocytes, recent evidence suggests that monocytes do not substantially contribute to macrophage populations in most tissues during normal homeostasis or certain inflammatory states [17]. Instead, it appears that macrophage populations within tissues maintain themselves predominantly by self-renewal, after being seeded in those locations by yolk sac precursors during embryonic development [18–20]. By contrast, tumor associated macrophages in the context of solid tumors [21–23] and inflammatory reactions associated with pathologic processes, such as atherosclerosis [24], are predominantly derived from circulating monocytes.

In addition, while self-renewal of resident macrophages may enable recovery in response to various forms of tissue injury (18), lethal radiation prior to bone marrow transplant (BMT) results in gradual replacement of tissue macrophages (with the exception of microglia) by donor monocytes in both mouse models [18, 25] and human patients [26, 27]. However, host macrophages may persist, particularly when fractionated doses of radiation are given, and be capable of repopulating the tissue [28]. Even when largely ablated, a small population of residual host macrophages may serve as antigen presenting cells and development of graft versus host disease [29].

The complex interplay between residual/recovering tissue macrophages and colonizing donor monocytes following BMT is of particular interest in recurrent CHL. Recurrent CHL often involves tissues with a large resident macrophage population such as lung and liver. Given the association with macrophage frequency and disease prognosis, we sought to further understand this process, including whether the inflammatory environment may change post-transplant. We characterize the inflammatory infiltrate in pre- and post-BMT tumor specimens in patients who had received an allogeneic BMT and investigate the origin of the inflammatory cells (donor or recipient) in the post-BMT specimens.

## Materials and Methods

Prior to commencing the study, approval was obtained from the Johns Hopkins Hospital Institutional Review Board (JHH IRB) under the IRB-approved protocol IRB NA_00083069. Per the JHH IRB, specific oral or written consent was not required for inclusion of specimens in this retrospective study, as the findings do not affect either diagnosis or treatment and both the diagnosis and treatment plans were established prior to and independent of the study findings. The pathology database was then searched to identify patients with CHL who were treated with an allogeneic BMT at our institution and subsequently developed relapsed disease. Clinical data were reviewed by one of the authors (A.S.D.) in accordance with IRB-approved protocol. Decalcified bone marrow biopsy specimens were excluded from the study.

Formalin-fixed and paraffin-embedded (FFPE) tissue was used to perform XY chromosome studies using fluorescence in situ hybridization (FISH; CEP X SpectrumOrange/Y SpectrumGreen Direct Labeled Fluorescent DNA Probe Kit, Abbott Molecular, Des Plaines, IL) in patients who received a sex mismatched BMT. Representative fields were chosen based on the corresponding H&E stained sections, and XY FISH images were captured with a fluorescence microscope. XY FISH was also performed subsequent to standard immunohistochemical staining for CD68 on the same slide.

Molecular chimerism studies (AmpF1STR Identifiler, Applied Biosystems, Foster City, CA) were performed on the post-transplant FFPE tissue in 3 of the 4 patients. This system uses PCR and capillary electrophoresis (ABI 3130xl, Applied Biosystems, Foster City, CA) to determine the length of 15 tetranucleotide short tandem repeats (STRs) in order to generate a molecular “signature” for the patient and donor. The relative percentages of donor and patient DNA in the post-BMT CHL samples were determined using the patient and donor STR profiles that were performed prior to the BMT.

The FFPE tissue was also subjected to hematoxylin and eosin (H&E) staining as well as immunohistochemical staining using routine methods for clinical diagnosis. All antibodies were from Ventana (CD15, 760–2504; CD30, 790–2926; CD68, 790–2931; CD20, 760–2531; Tuscon, AZ) with the exception of CD3 (ORG-8982, Leica, Bannock Burn, IL).

## Results

Four patients were identified that had relapsed CHL after an allogeneic BMT and also had post-BMT FFPE tissue involved by CHL available in our archives (Table 1). Two of these patients also had FFPE tissue available that was involved by CHL from before the BMT (Patients A and B).

**Table 1:** Demographic information.

	A	B	C	D
**Sex & Age at Initial Dx**	M; 31	M; 30	M; 34	F; 25
**Date, Dx & Site**	8/2007*, NS CHL, Stage IIIB, Cervical nodes, EBV(-)	7/2002*, NS CHL, Stage IIIB, Iliac node, EBV(-)	9/1995, CHL, Stage IIIAS, Cervical node, EBV(-)	10/2008, NS CHL, Stage IVB, Cervical node and marrow, EBV(+)
**Treatment**	9/2007, Rituximab-ABVD	7/2002, ABVD	9/1995, ABVD, splenectomy	10/2008, ABVD with omission of bleomycin
**Date & Site of 1**^**st**^ **Relapse**	3/2008, Axillary node (Primary refractory disease)	11/2003, Bone (sacrum)	10/1996, Cervical node	Primary refractory disease
**Treatment after 1**^**st**^ **Relapse**	4/2008, ICE	1/2004, ESHAP	4/1997, Conditioning regimen & BMT	4/2009, R-ICE, partial response, BMT
**BMT**	7/2008, Haplo-identical non-myeloablative, Donor: mother	3/2004, HLA-identical T-cell-depleted allogeneic bone marrow transplant, Donor: brother	4/1997, HLA-identical T-cell-depleted allogeneic bone marrow transplant, Donor: sister	6/2009, HLA-identical T-cell-depleted allogeneic bone marrow transplant, Donor: brother
**Date & Site of 2**^**nd**^ **Relapse**	12/2009, Portal Node and Liver	4/2006, Mediastinal Nodes	2/2008, Inguinal Node	3/2010, Lung
**Treatment after 2**^**nd**^ **Relapse**	Etinostat, brentuximab, ipilimumab	Rituximab, donor lymphocyte infusion	HDAC, Donor lymphocyte infusion, bendamustine, rituximab	Clinical trial with entinostat, disease progression, bendamustine
**Date and Site of 3**^**rd**^ **Relapse**	5/2015, Radiographic evidence, gemcitabine/ navelbine/ doxil, Nivolumab		6/2011, Lung & paratracheal nodes	
**Outcome**	4^th^ Recurrence, Axillary Node	Disease-related death	Disease-related death	Disease-related death

Pre-BMT biopsies in bold (Patients A and B, ‘*’) were characterized by immunostains. Post-BMT biopsies that were characterized are indicated by shaded boxes. The post-BMT biopsy for Patient A was characterized with immunostains, FISH and identity testing; the post-BMT biopsy for Patient B was characterized by immunostains and identity testing; the post-BMT biopsy for Patient C was characterized by FISH and identity testing and the post-BMT biopsy for Patient D was characterized by immunostains and FISH. The 4^th^ recurrence specimen for patient A, an axillary node biopsy following checkpoint inhibitor therapy, was also evaluated (S4 Fig). Abbreviations: Nodular sclerosing classical Hodgkin Lymphoma (NS CHL), Epstein-Barr Virus (EBV), R (rituximab)-ABVD (doxorubicin, bleomycin, vinblastine, dacarbazine), ICE (ifosfamide, carboplatin, etoposide), ESHAP (etoposide, methylprednisolone, high-dose cytarabine, and cisplatin) and HDAC (high dose cytarabine).

Three patients had undergone myeloablative transplants (Patients B, C and D), and one patient (Patient A) had a non-myeloablative (reduced intensity) transplant. Three of the four patients had opposite sex donors: patients (Patients A and C) were male but had a female donor and patient (D) was a female but had a male donor. Two of the patients (B and D) had bone marrow chimerism studies available near the time of relapse. In patient B, studies performed both one month before relapse and one month after relapse (but before the initiation of treatment) showed approximately 10% patient DNA and 90% donor DNA. Patient D had 100% donor DNA with no patient DNA detected both 8 months prior to relapse and 2 months after relapse.

**Fig 1:**
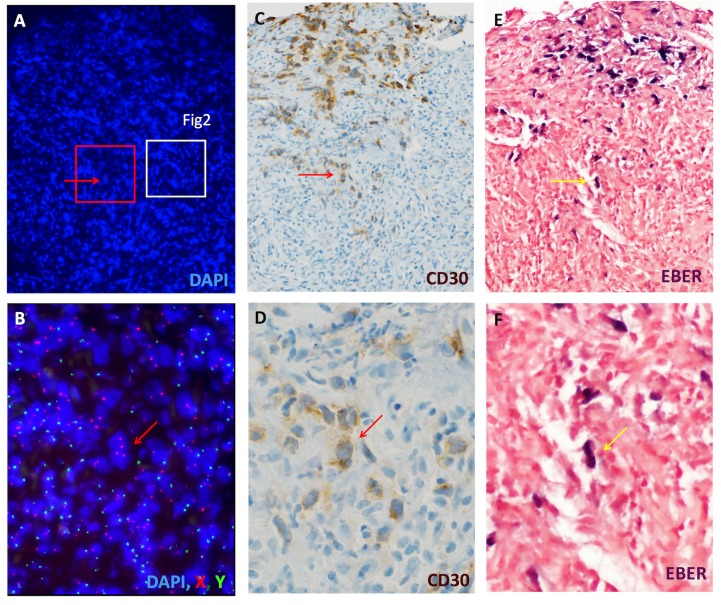
H/RS cells are patient-derived while the majority of the inflammatory infiltrate is donor-derived. Sections of lung from a female patient (Patient D) with recurrent Hodgkin lymphoma following an allogeneic BMT (brother) are shown. An overview of one of the lesional areas is shown (A, DAPI nuclear staining). XY FISH was performed on this section, and a high power view of the boxed area in red is shown in B (X = red, Y = green). An adjacent tissue section was stained for CD30 (C), highlighting numerous H/RS cells (arrow, higher magnification, D). While not possible to align perfectly, the H/RS cells in this patient were positive for EBV (in situ hybridization for EBER, panels E, F). The majority of the smaller nuclei are donor-derived (XY, red and green, 78% including only DAPI-positive nuclei with distinct FISH signals). By comparison with the H&E, these cells predominantly represent an inflammatory infiltrate. A separate area from this specimen demonstrating similar findings is shown in S1 Fig.

**Fig 2:**
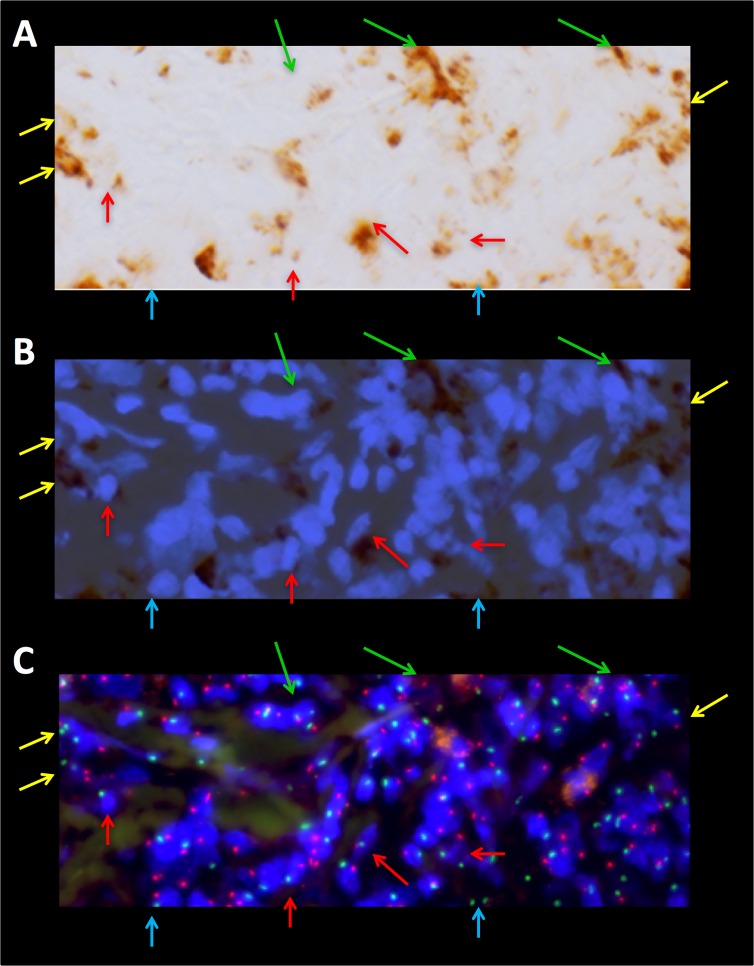
Tumor-infiltrating macrophages in recurrent Hodgkin lymphoma are predominantly donor- and, therefore, bone marrow-derived. A portion of the same field highlighted in Fig 1A (white box) is shown at higher magnification. Prior to analysis by XY FISH, the same slide was stained for CD68 using standard immunohistochemical techniques in order to identify macrophages (A). The images of the DAPI nuclear stain and the CD68 cytoplasmic stain are overlaid (B) to better identify individual cells in the corresponding FISH images (C). Where possible to discern, the tumor-infiltrating macrophages are all derived from the male donor (arrows, XY, red and green). However, by this method we found that in 21 +/- 4% of DAPI-positive nuclei, it was not possible to score X, Y status due to sectioning and/or other technical limitations. Of note, this patient had 100% donor chimerism in her bone marrow when tested near the time of this biopsy. H/RS cells (Fig 1 and S1 Fig) and areas of residual, uninvolved lung tissue were female (not shown). An overview of the density of macrophage infiltrate is shown in S2 Fig.

To assess the origin of the inflammatory infiltrate in the recurrent lymphoma we performed XY FISH on post-BMT tumor from patients A, C and D. Samples from all three patients demonstrated that the inflammatory infiltrate was largely composed of donor derived inflammatory cells (Fig 1, Fig 2, S1 Fig and S3 Fig). Scattered larger cells derived from the patient were present, which were compatible with HRS cells based on morphology and evaluation of adjacent sections stained for CD30 (Fig 1 and S1 Fig).

**Fig 3:**
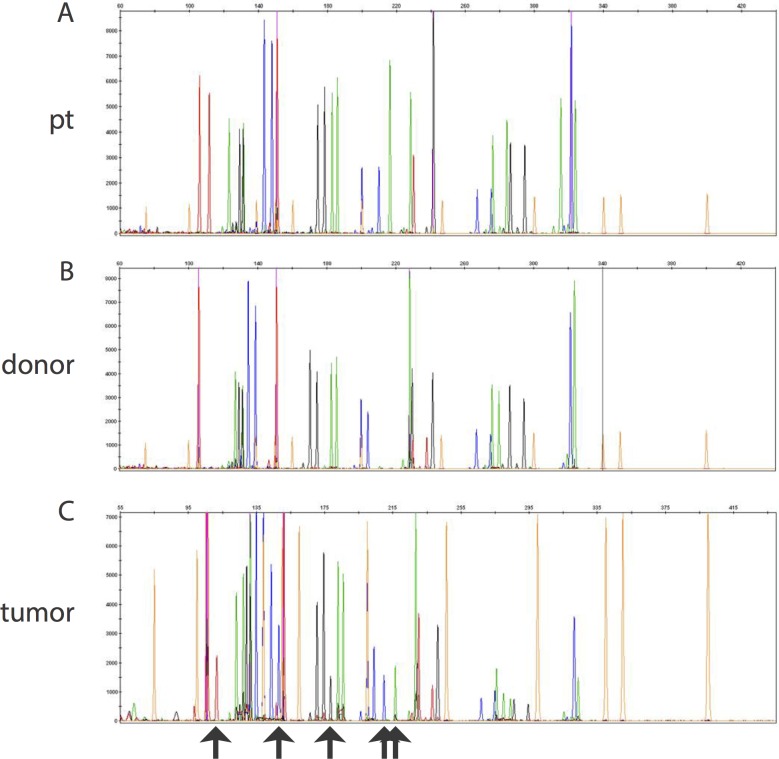
Molecular diagnostic studies show that the majority of cells in post-BMT tumor are derived from the donor. Molecular chimerism studies of pre- and post-BMT tumor (Patient C) are shown. Patient and donor specific peaks are shown in (A) and (B), respectively. Specific peaks derived from the patient DNA are highlighted by black arrows in the post-BMT tumor specimen (C). At the time of relapse this tumor specimen was 62% donor.

To confirm the FISH findings as well as to investigate the composition of the tumor in the patient with a same-sex donor (Patient B), molecular chimerism studies were performed on 3 of the 4 patients (excluding patient D). For all three, the molecular identity testing showed that the majority of the cells in the tumor were of donor origin (Patient A 89% donor; Patient B 72% donor; Patient C 62% donor) (Fig 3).

**Fig 4:**
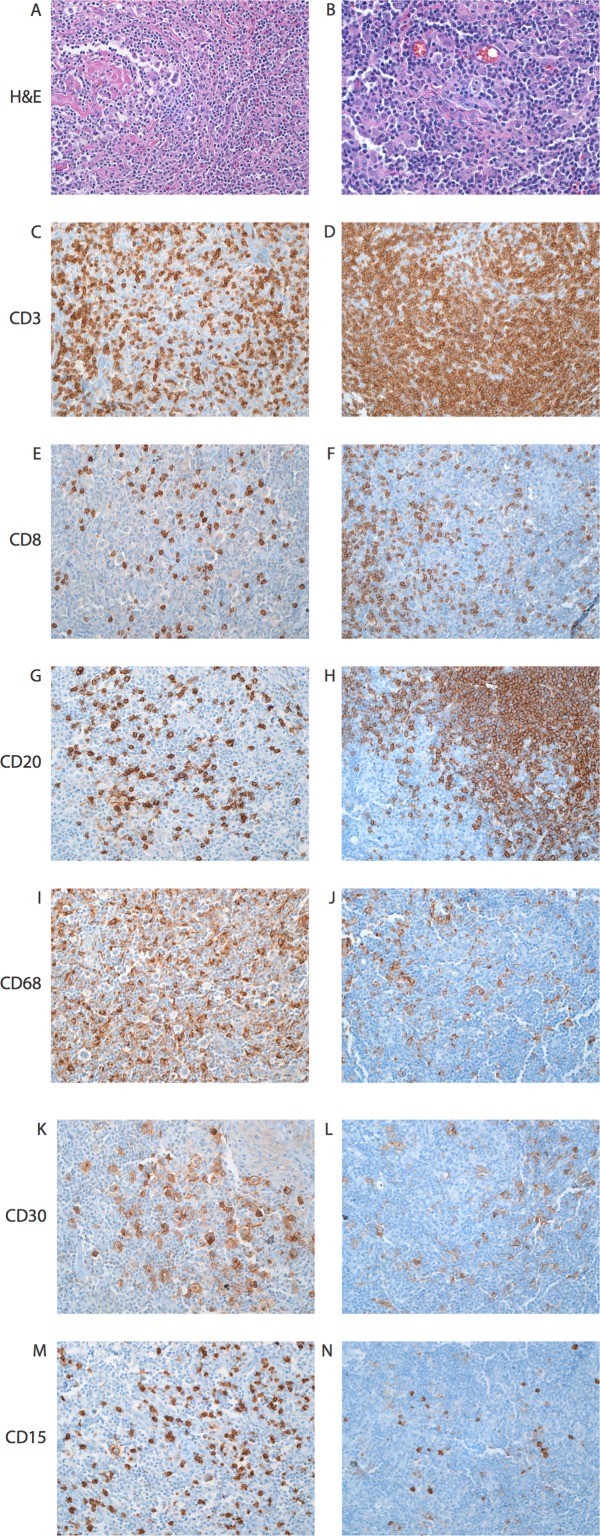
The composition of the inflammatory cells in pre and post-BMT specimens is similar. Representative high power fields from cervical (pre-BMT) and portal (post-BMT) lymph nodes involved by CHL from Patient A. Shown are H&E (A, B), CD3 (C, D), CD8 (E, F), CD20 (G, H), CD68 (I, J), and CD30 (K, L) for the pre-BMT sample (left) and the post-BMT sample (right) (200x).

For the two patients where pre- and post-BMT tissue was available (Patients A and B), IHC stains were performed to visualize the neoplastic cells and the composition of the inflammatory infiltrate (Fig 4). The percentage of CD68-positive macrophages, CD3-positive T-cells, CD8-positive T-cells, and CD20-positive B-cells was estimated by 5 experienced hematologic pathologists (M.V.-R., C.D.G., K.H.B., M.J.B., and A.S.D.) who did not know the identity of the specimens. Both patients’ specimens showed a similar composition of CD68-positive macrophages and CD8-positive T-cells as a percentage of total T-cells in the pre- and post-BMT tumor (Table 2). In both patients B-cells were more numerous in the post-BMT sample, although the significance of this finding is unclear. Notably, the initial treatment for patient A included rituximab prior to BMT. The inflammatory infiltrate was also characterized for patient A at the time of the 4^th^ relapse, though the patient had been treated with the anti-PD-1 monoclonal antibody nivolumab prior to the biopsy (S4 Fig).

**Table 2:** Composition of the Inflammatory Infiltrate.

Sample	CD68+ Cells	CD8+ T-cells	CD3:CD20 ratio
pt A pre	20%	15%	6.5:1
pt A post	15%	20%	1.7:1
pt B pre	10%	67%	8.4:1
pt B post	5%	67%	1:2.2

Semi-quantitative measurements of CD68-positive macrophages, CD8-positive T-cells, and the CD3 to CD20 ratio for the pre- and post-BMT samples from two patients with available pre-BMT CHL tumor specimens are shown. The provided values are the median of semi-quantitative estimates from five hematologic pathologists.

## Discussion

In this study we utilized post-BMT tumor specimens in order to more fully characterize the relationship between H/RS cells and the tumor-associated inflammatory cells, in particular the origin of the tumor-associated macrophages. These findings support the idea that H/RS cells, perhaps through cytokines and chemokines, actively recruit and define the inflammatory microenvironment [30–35]. In particular, the H/RS cells appear to recruit donor-derived circulating monocytes from the bone marrow as opposed to stimulating the proliferation of resident macrophages within the tissue. For example, in Patient A, FISH showed only rare cells with patient DNA while tumor-infiltrating macrophages comprised about 15% of the cellularity in the node (Fig 4 and S3 Fig). Macrophages that were donor-derived could also be readily identified by co-staining the same slide for CD68 by immunohistochemistry and XY FISH (Patient D, Fig 2) and appeared to comprise the vast majority of the macrophages within the lesion.

Additionally, we found that the composition of the inflammatory infiltrate was very similar in the pre- and post-BMT samples where evaluable. The percentage of macrophages for the two patients examined in this manner fell into the “intermediate risk” category as defined by Steidl, et al. with between 5 and 25% macrophages [13] and similar percentages in the relapsed specimens post-BMT. The percentage of CD8-positve T-cells also remained similar pre- and post-BMT, suggesting that the H/RS cells are recruiting a specific complement of inflammatory cells. Of note, EBV may affect the inflammatory environment by altering cytokine expression [36] and has been associated with increased CD8+ T-cells, increased LAG-3-positive regulatory T-cells and increased macrophage recruitment [11, 37]. However, in this series, only one of the four cases was EBV-associated, and a corresponding primary diagnostic specimen was not available for comparison in this patient. Even in the absence of EBV, studies of the inflammatory environment in CHL have demonstrated an association with immunosuppressive molecules and an impaired T-cell response [37, 38]. For example, increased expression of immune modulators such as indoleamine 2,3-dioxygenase (IDO) was associated with worse clinical outcome. IDO expression strongly correlated with extent of macrophage infiltration and was expressed by macrophage/monocyte-derived cells [38]. While it is possible that BMT may result in an alteration in the tumor microenvironment to a more pro-inflammatory state with graft versus tumor effect, the similar inflammatory environments pre and post-transplant in terms of percent of tumor-infiltrating macrophages, and fractions of CD4 and CD8+ T-cells argue against this possibility. Nonetheless, this does not rule out the possibility that some patients may have a favorable change in their immune response post-transplant.

One potential limitation on the generalizability of this study for understanding tumor-infiltrating macrophage recruitment to the CHL microenvironment is the demonstration in prior studies that total body irradiation is associated with gradual replacement of tissue macrophages by bone marrow-derived monocytes [18, 25–27]. However, our study did include one patient who had undergone a non-myeloablative BMT (Patient A), and even in that patient, the inflammatory infiltrate remained predominantly donor-derived (S3 Fig). This suggests the recruitment of bone marrow derived monocytes to recurrent CHL is not a mere artifact of the BMT conditioning regimen.

While the number of cases is small, our findings raise the possibility of novel treatment strategies for recurrent CHL, including the potential to pharmacologically inhibit monocyte recruitment to tumor lesions to slow or alter disease progression [39,40].

## Supporting Information

S1 Fig Large atypical cells consistent with H/RS cells are patient-derived in recurrent Hodgkin lymphoma following BMT. A separate area of recurrent CHL involving the lung from patient D is shown (compared to Fig 1). XY FISH revealed that large atypical nuclei were predominantly derived from the female patient (XX, red). In addition, there were smaller patient-derived nuclei (XX, red) with a distribution consistent with endothelial cells. However, the majority of the surrounding inflammatory cells appeared donor-derived (XY, red and green). This corresponded to 78% of total nuclei (as highlighted by DAPI), including only cells where possible it was possible to identify distinct FISH signals. Adjacent sections show H/RS cells by CD30 staining and histomorphology (H&E) with a similar distribution to the large atypical, patient-derived cells.
(TIF)

S2 Fig Numerous macrophages are present with the inflammatory infiltrate of recurrent Hodgkin lymphoma. The same image shown in Fig 1A is displayed for orientation (A), representing recurrent CHL involving the lung in Patient D. The corresponding CD68 immunostain of the same tissue section (B) reveals numerous tumor-infiltrating macrophages throughout the lesion comprising approximately 10–20% of the cellularity.
(TIF)

S3 Fig ISH studies from a patient with a non-myeloablative (reduced intensity) BMT. **Even in the reduced intensity BMT setting, the majority of cells in the recurrent tumor are donor-derived.** XY FISH studies of the pre- and post-BMT tumor from Patient A are shown. Nuclei are stained with DAPI (blue). Red and green signals correspond to the probes on the X and Y chromosomes, respectively. The pre-BMT tissue (cervical lymph node) demonstrates both X and Y chromosomes in all cells whereas the post-BMT tissue (portal lymph node) shows that most cells are XX (red; donor, 85% of those that could be identified) with only scattered large XY (red and green; patient, 15% of those that could be identified) cells. In 21% of nuclei, the X, Y FISH probes could not be definitively evaluated due to the plane of section.
(TIF)

S4 Fig Inflammatory cells in a post-BMT specimen status post checkpoint inhibitor therapy. Representative high power fields from an axillary lymph node involved by CHL from Patient A post-BMT and post-treatment with the anti-PD-1 monoclonal antibody nivolumab (4^th^ relapse; 200x). There is a suggestion of a decrease in the percentage of CD8+ cells as compared to both the initial pre and post-BMT specimens, but the findings are otherwise similar. All antibodies were from Ventana (Pax-5, 790–4420; CD4, 790–4423; CD8, 790–4460; PD-1, 760–4895; Tuscon, AZ), with the exception of lymphocyte-activation protein 3 (Lag3, 17B4; LS Bio, Seattle, WA) and indolemine 2,3-dioxegenase (IDO, AB 9900, Millipore, Billerica, MA). IDO is visualized as red staining.
(TIF)
