# Supplementary figures and images for: Tumor-Infiltrating Macrophages in Post-Transplant, Relapsed Classical Hodgkin Lymphoma Are Donor-Derived
Source: PLoS One. 2016 Sep 29;11(9):e0163559. doi: 10.1371/journal.pone.0163559 (PMC5042490; doi:10.1371/journal.pone.0163559)

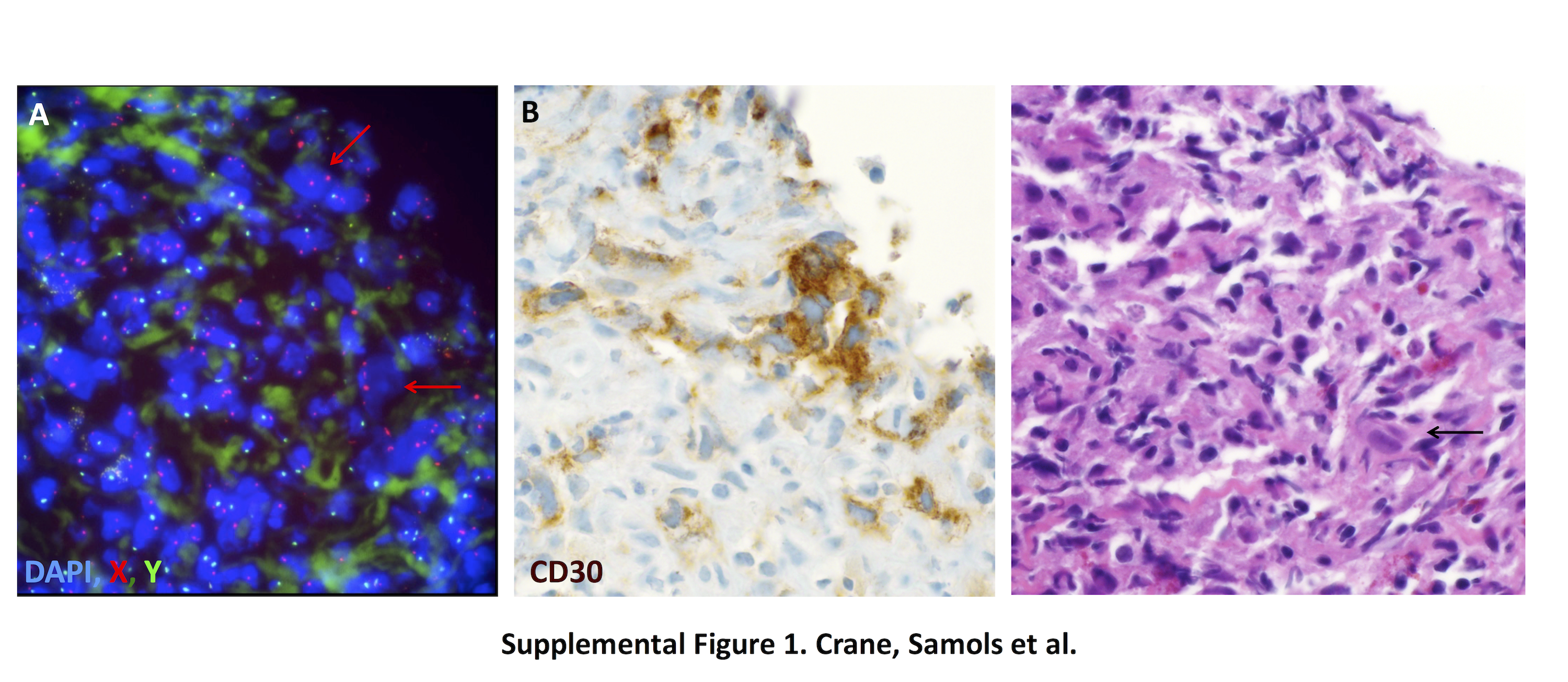

Supplement: S1 Fig — A separate area of recurrent CHL involving the lung from patient D is shown (compared to Fig 1). XY FISH revealed that large atypical nuclei were predominantly derived from the female patient (XX, red). In addition, there were smaller patient-derived nuclei (XX, red) with a distribution consistent with endothelial cells. However, the majority of the surrounding inflammatory cells appeared donor-derived (XY, red and green). This corresponded to 78% of total nuclei (as highlighted by DAPI), including only cells where possible it was possible to identify distinct FISH signals. Adjacent sections show H/RS cells by CD30 staining and histomorphology (H&E) with a similar distribution to the large atypical, patient-derived cells. (TIF) [file pone.0163559.s001.tif]

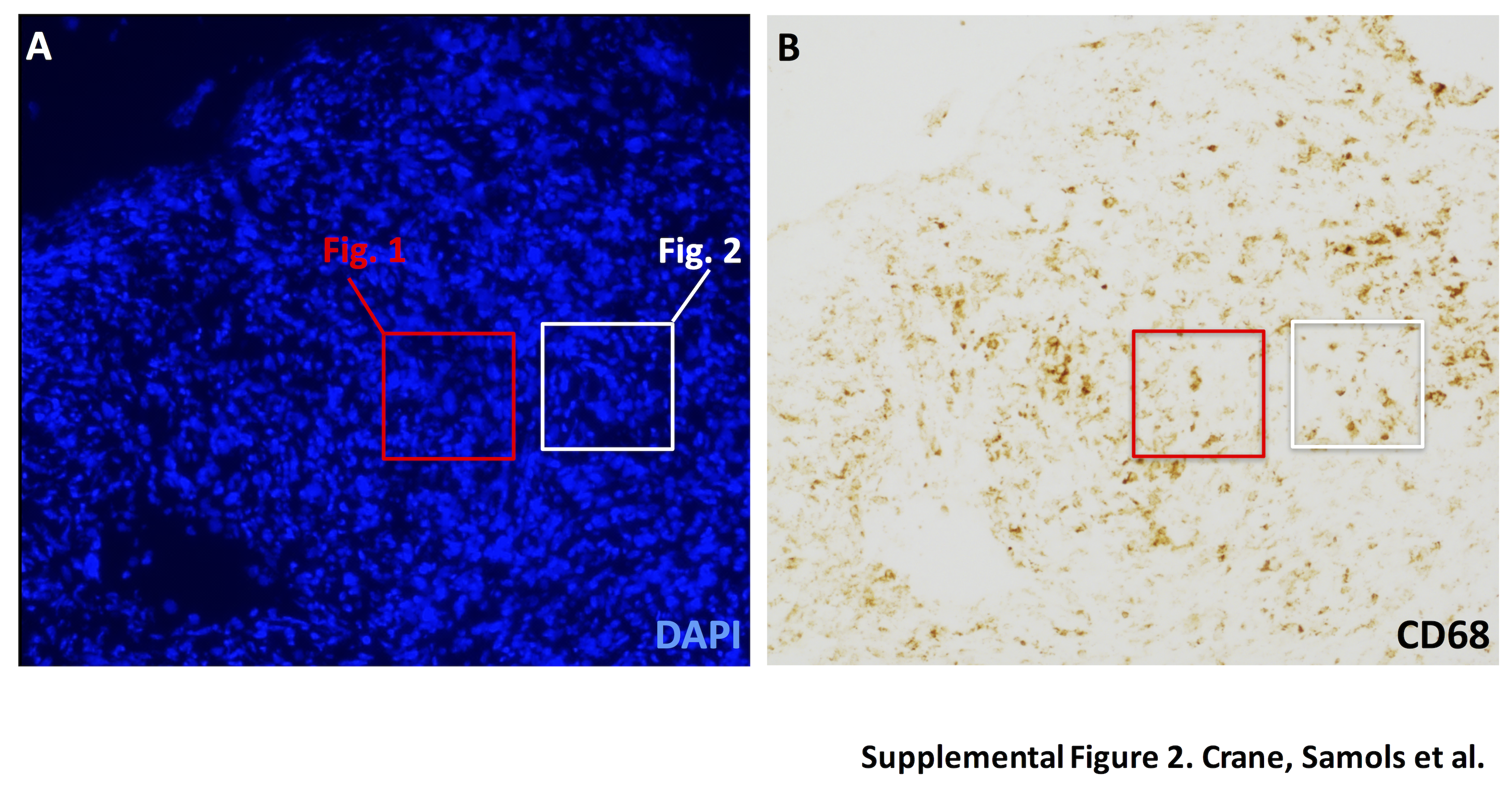

Supplement: S2 Fig — The same image shown in Fig 1A is displayed for orientation (A), representing recurrent CHL involving the lung in Patient D. The corresponding CD68 immunostain of the same tissue section (B) reveals numerous tumor-infiltrating macrophages throughout the lesion comprising approximately 10–20% of the cellularity. (TIF) [file pone.0163559.s002.tif]

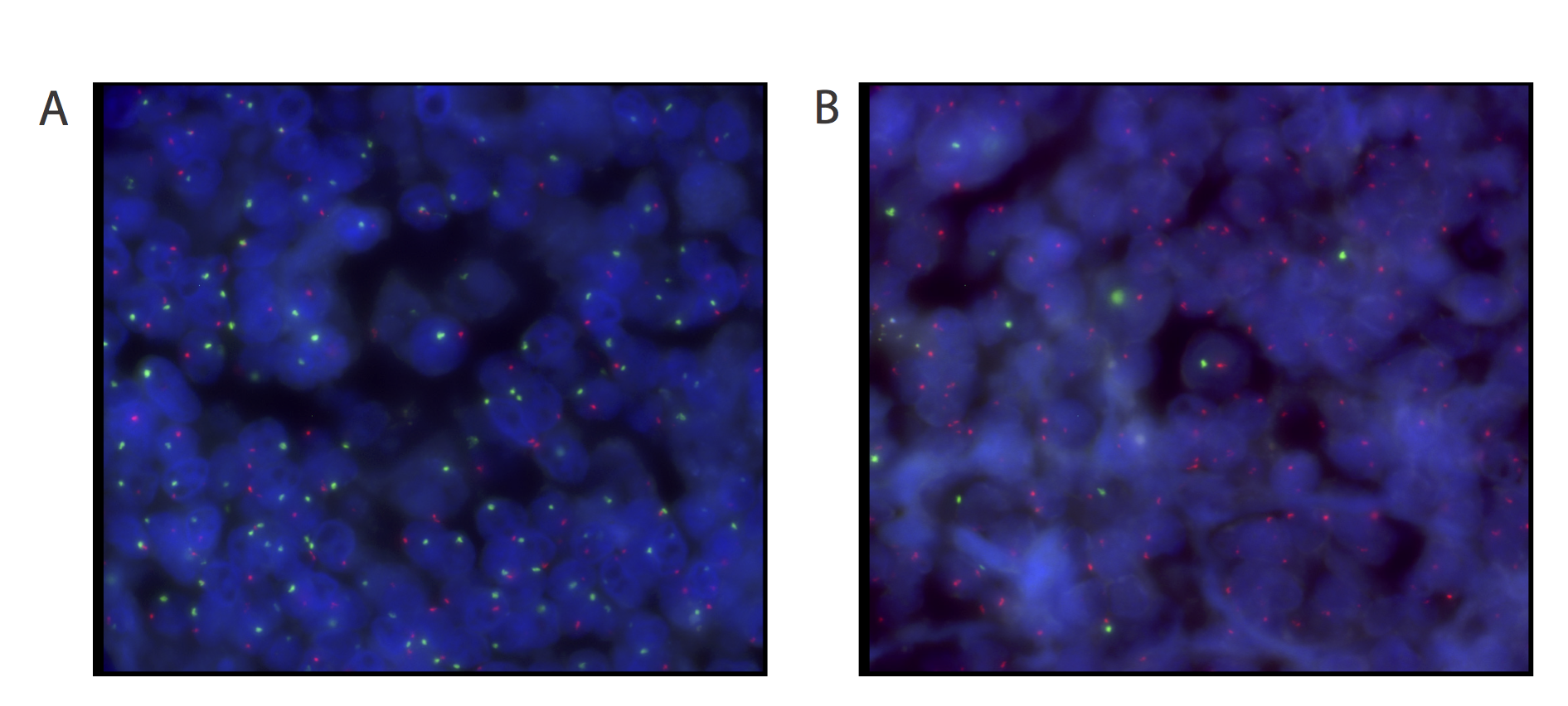

Supplement: S3 Fig — Even in the reduced intensity BMT setting, the majority of cells in the recurrent tumor are donor-derived. XY FISH studies of the pre- and post-BMT tumor from Patient A are shown. Nuclei are stained with DAPI (blue). Red and green signals correspond to the probes on the X and Y chromosomes, respectively. The pre-BMT tissue (cervical lymph node) demonstrates both X and Y chromosomes in all cells whereas the post-BMT tissue (portal lymph node) shows that most cells are XX (red; donor, 85% of those that could be identified) with only scattered large XY (red and green; patient, 15% of those that could be identified) cells. In 21% of nuclei, the X, Y FISH probes could not be definitively evaluated due to the plane of section. (TIF) [file pone.0163559.s003.tif]

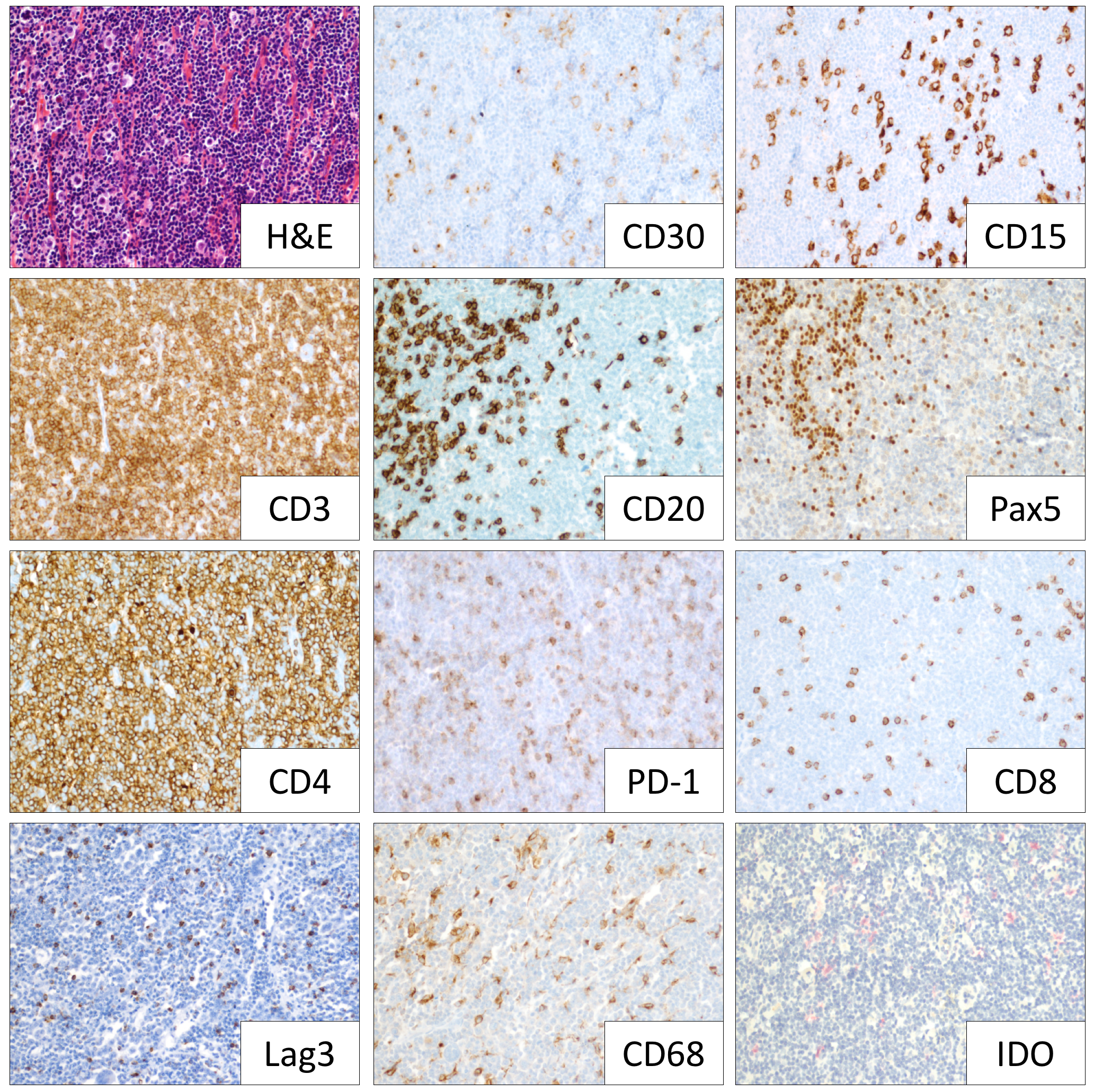

Supplement: S4 Fig — Representative high power fields from an axillary lymph node involved by CHL from Patient A post-BMT and post-treatment with the anti-PD-1 monoclonal antibody nivolumab (4th relapse; 200x). There is a suggestion of a decrease in the percentage of CD8+ cells as compared to both the initial pre and post-BMT specimens, but the findings are otherwise similar. All antibodies were from Ventana (Pax-5, 790–4420; CD4, 790–4423; CD8, 790–4460; PD-1, 760–4895; Tuscon, AZ), with the exception of lymphocyte-activation protein 3 (Lag3, 17B4; LS Bio, Seattle, WA) and indolemine 2,3-dioxegenase (IDO, AB 9900, Millipore, Billerica, MA). IDO is visualized as red staining. (TIF) [file pone.0163559.s004.tif]
